# O6.3. PATTERNS OF GRAY MATTER ABNORMALITIES IN PATIENTS WITH FIRST-EPISODE AND TREATMENT-NAïVE SCHIZOPHRENIA

**DOI:** 10.1093/schbul/sby015.224

**Published:** 2018-04-01

**Authors:** Mingli Li, Tushar Das, Deng Wei, Yinfei Li, Xiaohong Ma, Hua Yu, Xiaojing Li, Ya-jing Meng, Liansheng Zhao, Yingcheng Wang, Qiang Wang, Lena Palaniyappan, Tao Li

**Affiliations:** 1 Mental Health Center, West China Hospital of Sichuan University; 2 University of Western Ontario

## Abstract

**Background:**

To detect schizophrenia-related anatomical changes that are not confounded by antipsychotic treatment and to establish clinically identifiable subgroups that differ in underlying neuroanatomical patterns.

**Methods:**

This case-control study was conducted at West China hospital in China, and analysis was undertaken in Robarts Research Institute, London, Canada. 206 patients with schizophreniform psychosis and schizophrenia and 170 healthy controls were scanned on a Signa 3.0-T MR scanner; 137 patients with schizophreniform psychosis and schizophrenia and 172 healthy controls were scanned on a 3.0 T MR scanner. All the patients were ﬁrst-episode and treatment-naïve. Source based morphometry (SBM) performed to analyze the gray matter (GM) concentration. Latent class analysis used to identify clinical subtypes of patients using the scores of symptom dimensions. GMC component-based connectomes were constructed to study the graphic organization of structural brain network of subtypes of schizophrenia.

**Results:**

Patients showed prominent reduction in GM in two components; one including anterior insula, inferior frontal gyrus, anterior cingulate and another with superior temporal gyrus, and precuneus, inferior/superior parietal lobule, cuneus, and lingual gyrus. Increased GM was seen in one component of cerebellar tonsil and inferior semi-lunar lobule, and the other component of middle temporal gyrus, superior temporal gyrus, middle frontal gyrus and putamen. Greater GM of latter component was associated with less severe positive symptoms and better performance on cognitive tests. Reduced global efficiency only existed in a subgroup of patients with severe negative and disorganization symptoms.

**Discussion:**

These findings delineate a common pattern of gray matter changes in schizophrenia, and a subgroup of patients with robust cortical reorganization suggestive of compensatory plasticity after first episode.

